# The use of exemplars and student discussion to improve performance in constructed-response assessments

**DOI:** 10.5116/ijme.5b77.1bf6

**Published:** 2018-08-24

**Authors:** Fiza Rashid-Doubell, Paul A. O'Farrell, Salim Fredericks

**Affiliations:** 1 RCSI Bahrain, School of Medicine, The Royal College of Surgeons in Ireland, Adliya, Bahrain

**Keywords:** Medical students, constructed-response assessment, feedforward, short-answer questions

## Introduction

Assessment has a significant influence on learning, and different forms of assessment may have varying degrees of influence on students. Constructed-response assessments, i.e., those requiring a human to judge the quality of responses based upon criteria,^1^ differ from selected-response assessments, e.g., multiple choice questions (MCQs), which can be scored by a machine. Therefore, constructed-responses require the assessee to have some insight into the rationale and reasoning of the assessor and an appreciation of assessment criteria. However, students often struggle to get a sense of what constitutes a well-constructed or a poorly-constructed response to an examination-item.^2^ This problem is compounded by processes that require students to draw upon both explicit and tacit knowledge.^3^ Tacit knowledge being knowledge gained experientially and derived from shared understandings.^4^^,^^5^ These processes are often evident to teachers but imperceptible to students.^6^

Exemplars potentially provide a means of making complex marking criteria more comprehensible to students. Exposing students to examples of “real students’ work” of different standards has been shown to assist students in recognising writing-quality for themselves.^7^^,^^8^ Studies have shown exemplars to have been used as interventions for improving student performance,^4^^,^^7^ and as general tools for training students on how to write well-constructed responses.^3^  They have also been used as the basis of feedforward interventions^3^^,^^7^^,^^9^ i.e., anticipatory academic feedback given to students ahead of examinations. Thus, exemplars may be employed to improve responses of students to constructed-response assessment items such as short-answer questions (SAQs). Here we reflect upon an attempt to convey elements of academic reasoning and aspects of the assessment processes to students using exemplars. 

### Exemplars

Tutorials were used to present a number of exemplars to first-year medical students. The exemplars were responses to SAQs from past examinations and were identified as being clearly aligned with the faculty’s grade descriptors. Tutors introduced a 5-point closed marking scheme and described the rubric. Five biochemistry and five physiology exemplar SAQs were presented with grades ranging from 0 to 4. The tutors triggered discussions on how to analyse and score these items. Emphasis was placed on the essence of the questions being aligned with that of the responses. The discussions explored the structure for a good-quality response and common misconceptions about constructing responses to SAQs. Students were also asked to assign marks to ‘ungraded’ exemplars, thus allowing familiarisation with the grading process and academic reasoning.

The face-to-face tutorials were followed by online exercises that required the students to construct responses to a number of SAQs and to grade the responses of their peers, therefore providing the opportunity to receive feedback on their responses and to grade the responses of others. This exercise initiated online discussions about the whole process and underpinned the issues raised during the face-to-face tutorials. Thus, the exemplars became the initiators and influencers of several teacher-learner and learner-learner dynamic interactions.  Perspectives on these interactions are presented below.

### Perspective of students

The exemplars initiated dialogue between teachers and students. Since previous students wrote the exemplars, it showed current students the variation in writing capability. The importance of this has been previously reported upon and discussed by Scoles.^7^ The exemplars helped teachers articulate their standards and illustrate these standards to the students.^10^ These exemplars were seen as a component in a toolkit for assisting students with the assessment.^11^ The students agreed that it gave them insight into the marking process. However, our experience of using exemplars alone was that they were not sufficient to improve SAQ writing. Students also needed to practice writing and marking others in the online peer-assessment exercises for them to gain a better understanding of what constitutes a good answer and how to write one. The students also agreed that the online exercises gave them the opportunity to practice SAQs, as well as allowing them to see the other students’ responses to the questions. Students’ perceptions were gathered using an online questionnaire, which included a section for open-ended comments. Moreover, the teachers’ observations and reflections were used along with free-comments from the virtual student forum.

### Perspective of teachers

Students that participated in the tutorial and all aspects of the online activity achieved the best results in the summative assessment, relative to those that did not participate in all activities. However, we consider the engagement with the exemplars and the interactions with peers and teachers to be the most important aspect of this exercise, rather than examination results. Face-to-face and online discussions of the reasoning and logic involved in answering examination questions is beneficial for students on several levels. Discussion, reviewing and reflection stimulate metacognitive processes,^12^ which are key to accelerated learning. This process of test-enhanced learning encourages deep rather than surface learning, which may result from feedback and associated discussions, rather than the assessment itself.^13^ Draper suggests that accelerated learning is the result of stimulating thought and/or discussion with peers regardless of formal feedback from skilled teachers. The emphasis is placed on events following assessments that induce intrinsic metacognitive processes and extrinsic Socratic dialogues.^14^ Although it is reported that dialogue and discussion centred upon assessment items promotes learning, the mechanisms involved in triggering higher-order thinking processes seem poorly understood. This is an area requiring further research in the context of medical education.

Students seemed surprised at the grades allocated by teachers. These marks were lower than those assigned by students when they were invited to grade the exemplars. This mismatch provided a good launching point for discussions around the tacit understanding needed for students to determine the quality of responses shown. During the teacher-led discussion, deficiencies in the weak exemplar answers emerged, as students discovered for themselves the missing elements and poor approaches to answering those questions. Some students expressed surprise at the level of detail contained in higher scoring answers. Here, the use of exemplars was crucial, showing students what was possible to be produced by one of their peers under examination conditions. Showing high scoring exemplars also emphasised that an answer produced by a student does not have to be a verbatim copy of the model answer used for grading in order for it to score well. This allowed students to see that scoring and grading was the product of a human intellectual process and not the product of a pre-programmed optical recognition device. 

Potentially, this use of exemplars in the two settings (face-to-face and online) exposed students to the whys and wherefores of constructed-response assessments in medical education. Clinical, practical and theoretical aspects of medicine are assessed using various guises of constructed-responses, while the selected-response items such as multiple-choice questions are often confined to assessing rote-learned knowledge. Many types of assessments experienced by healthcare professionals, at all levels, are scored by a human^1^ rather than a machine. Healthcare professionals must therefore acquire reasoning, logic and an ability to structure coherent responses in order to measure up to a standard which is judged by another person. If students can grasp the basic thought processes necessary to construct a response to an SAQ, then they may also grasp the common thought processes involved in responding to performance-based (clinical assessments) and other paper-based tests. Academic reasoning and clinical reasoning have common foundations but may differ in their detail. The acquisition of techniques for systematic and logical thought processes is critical to performance. This is applicable in both the clinic and the examination hall.

## Conclusions

It is possible to improve academic performance using exemplars in tutorials. Face-to-face interactions encourage necessary discussion between teachers and learners, as well as between learners and learners. These interactions are invaluable for augmenting cognition of writing quality. However, as an essential adjunct to the traditional tutorial; students should address the issues of quality and assessment-criteria in their space and among themselves. These separate student-student interactions with the subject further facilitate awareness of the cognitive skills needed to construct responses of high quality. It is this additional student-led discussion around these issues that may be a key component of success in examinations. This notion of students discussing assessment with their peers becomes important within the broad remit of internationalising medical education. Programmes are often delivered in English to students whose native language is not English. Peer-led deliberations may be conducted in a common vernacular other than Standard English, while allowing high-level cognitive processes to remain in effect. The context of the discussion will always be framed within the requisite exemplar. Essentially, exemplars are necessary tools to provide more explicit context when working to globalise medical education. Student-led interactions may result in more generalised improvements in performance in the many constructed-response assessments employed in medical education. 
